# Comparison of modified Bass, Fones and normal tooth brushing technique for the efficacy of plaque control in young adults- A randomized clinical trial

**DOI:** 10.4317/jced.55747

**Published:** 2020-02-01

**Authors:** Chandrashekar Janakiram, Naveen Varghese, Ramanarayanan Venkitachalam, Joe Joseph, Karuveettil Vineetha

**Affiliations:** 1 Professor and Head, Department of Public Health Dentistry. Amrita School of Dentistry, Amrita Vishwa Vidyapeetham, AIMS, Ponekkara P. O., Kochi 682041

## Abstract

**Background:**

To compare the anti plaque efficacy of Modified Bass, Fones and Normal brushing techniques in young adults.

**Material and Methods:**

An investigator blinded randomized controlled trial with parallel design was adopted to compare the anti plaque efficacy of three tooth brushing techniques. The study population consisted of 120 dental students aged between 18 and 30 years.

**Results:**

At the baseline, the mean plaque scores were 0.74 ± 0.39, 0.77 ± 0.34 and 0.98 ± 0.36 respectively, for Modified Bass, Fones and Normal brushing technique. After 24 hours without any oral hygiene activity, the plaque scores increased to 1.04 ± 0.30, 1.11 ± 0.32 and 1.21 ± 0.40, respectively. After 1 week of using the intervention, the mean plaque scores were 0.78 ± 0.36, 0.94 ± 0.34 and 1.03 ± 0.43, respectively and increased to 1.13 ± 0.44, 1.14 ± 0.40 and 1.08 ± 0.34 after 28 days. The mean gingival scores were 0.23 ± 0.66, 0.02 ± 0.52 and 0.42 ± 0.74 for Modified Bass, Fones and Normal Brushing technique during baseline visit and after 28 days.

**Conclusions:**

There was a significant reduction in the amount of plaque with the three brushing techniques. Although the short-term outcomes with the Modified Bass method were promising, a long-term effect was not evident. Further, there was no significant difference in plaque control between the three groups.

** Key words:**Gingival scores, plaque scores, tooth brushing techniques, young adults.

## Introduction

The prevalence of oral diseases is high in India with dental caries (79.2%, and 84.7% in 35-44, and 65-74 year old, respectively) and periodontal diseases (89.2%, and 79.4% in 35-44, and 65-74 years old, respectively) being the two most commonly reported oral entities (1). Plaque is the single most important cause of dental caries and gingival diseases. It is described as a soft, tenacious material found on tooth surfaces, which is not readily removed by rinsing with water (2). Plaque found below the gingival margin in the gingival sulcus or in the periodontal pocket is termed sub gingival plaque (2).

The effect of plaque control on gingivitis and periodontitis is well-documented and tooth brushing twice daily along with the use of other oral hygiene aids, prevents the initiation of gingivitis as well as its progress. Gingivitis can be noted within a few days of stopping oral hygiene practices (3). Tooth brushing is the most widely accepted mechanical means of plaque control due to its effectiveness, convenience, as well as low cost (4).

The various tooth brushing techniques practiced are Roll or Modified Stillman, Stillman’s, Charters, Bass, Modified Bass, Fones, Leonard, and Scrub. Modified Bass technique was reported to be the most effective brushing technique followed by Horizontal Scrub technique, while the least effective was Fones (3). This was based on improved plaque control and reduced gingival inflammation with modified Bass and horizontal scrub techniques compared to others. However, these findings were based on a small study population and short follow up periods. Modified Bass technique was also reported to be superior in reducing supragingival plaque than Normal tooth brushing practices (5). An optimal reduction in plaque with adequate protection of the oral tissues against mechanical trauma is achieved by manual tooth brushing with the use of Modified Bass technique (6).

However, there is a report suggesting that Fones technique was efficient in reducing the amount of plaque (7) while in few other studies the Horizontal technique (Normal Brushing technique) was found to be most effective (8-10) with the advantage that it is easier to implement (11,12). On the contrary, many studies show that there were no significant differences between various brushing techniques with regard to their plaque removal effectiveness (13-16). Though the Modified Bass and Scrub method have been most commonly recommended, there are only very few evidence available to substantiate the choice of one technique over the other (17).

Hence, there exists conflicting results in different studies conducted regarding which brushing technique is better and hence there is an urgent need for research into the comparative effectiveness of different brushing methods (18). So a study was devised with the objective to compare the anti-plaque efficacy of Modified Bass, Fones and Normal brushing technique in young adults.

## Material and Methods

-Study population and methodology

This was a randomized controlled trial with parallel design to compare the anti plaque efficacy of three different tooth brushing techniques (1:1:1 ratio). The different brushing techniques used were Modified Bass, Fones and Normal brushing technique. In this study Normal brushing technique implied the usual method followed by the study participants. The study participants were young adults aged 18-30 years and was recruited from a dental school in India. This population was chosen as the study sample as the trial required multiple follow-ups and strict adherence to the protocol. Ethical approval was obtained from the Institutional Review Board. The trial was registered with the Clinical Trial Registry of India (Reg No: CTRI/2017/12/010815) under the web site (http://ctri.nic.in).

-Selection criteria

Patients with mild to moderate gingivitis having at least 20 natural teeth with no history of periodontal therapy or antibiotic medication preceding 6 months from the start of study were selected. Voluntary informed consents were obtained from the participants. Patients undergoing orthodontic treatment, having advanced periodontal disease, pregnancy or women who are breast-feeding, those who received a dental prophylaxis in the past two weeks prior to the baseline examinations and smokers were excluded from the study.

-Organizing the study

Eligibility was assessed by the principal investigator and the participants were detailed regarding the procedures to be conducted during each visit. Written voluntary informed consent was obtained from willing participants. Since the principal investigator was blinded to the intervention, a trial coordinator was appointed for the randomization, allocation of intervention and demonstration of tooth brushing technique. A trial register was also maintained to record the date and time of each visit. All the outcomes were assessed by a single examiner. Before and during the study the examiner was trained /re-calibrated. Every 10th patient was reexamined for reliability. The examiner was calibrated for recording the Plaque index and Kappa value was 0.845.

-Intervention

Each eligible participant was randomly assigned to either one of the three groups i.e. Modified Bass technique, Fones technique and Normal tooth brushing technique using computer generated randomization sequence and randomized accordingly with the help of the trial coordinator to each intervention.

a. The active arm consisted of brushing twice daily with Modified Bass and Fones technique using tooth paste and a tooth brush. The reciprocal arm consisted of brushing twice daily with Normal brushing technique with tooth paste and tooth brush. A standard tooth brush (soft bristled) and tooth paste (Colgate Advanced Health) 70 grams were used for all participants included in the study. Participants were advised to load the toothpaste till one half of the toothbrush before brushing. This was done to ensure that one gram (equal quantity) of toothpaste was used each time. The received intervention (brushing technique) was performed by the participants in the presence of the trial coordinator during each visit to ensure proper implementation. The brushing method for Modified Bass and Fones technique were demonstrated by the trial coordinator using power point slides and videos. Study models were also used for demonstration. In order to ensure compliance of brushing technique, a leaflet describing the steps in performing the prescribed brushing technique was provided to the participants. They also received a Short Messaging Service (SMS) alert daily in morning and night. The participants were also asked to demonstrate prescribed technique during their re-visits at random.

-Outcomes Evaluated 

The outcome evaluated was the reduction in plaque and gingival scores during each visits between the groups. This was measured during four visits (Visit 1 –Baseline, Visit 2 – 24 hours without brushing, Visit 3- one week later and Final Visit – 28 days later) from the patient. Plaque scores were assessed using the Turesky-Gilmore-Glickman modification of Quigley Hein plaque index. A disclosing agent was used (AlphaPlac, Two-Tone disclosing agent, DPI, Mumbai) prior to the assessment of plaque scores. Gingival index developed by Loe H and Sillness *P* were used to assess the severity of gingivitis. William’s periodontal probe was used to assess the bleeding.

-Sample size

Sample size was estimated using nMASTER software based on results from a similar study done by Hernacke *et al.* (19). The following parameters were used to calculate the sample size.

• Standard deviation (Fones technique)=0.55

• Standard deviation (Normal brushing technique)= 0.44

• Mean difference= 0.32

• Effect size=0.64

• Alpha error=5%

• Power of study=80%

• Required sample size= 38 in each group

• Final sample size: 40 + 40 + 40 = 120

-Statistical analysis

Statistical Package for Social Sciences (SPSS, Version 18) software was used for the analysis. The buccal, lingual and total plaque and gingival scores of each visit were expressed as mean and standard deviation. The results of oral hygiene practices were expressed in the form of frequencies. Within group comparison of mean gingival scores during follow up visits was done using paired t-test. Analysis of Variance (ANOVA) was applied to know whether there was any statistically significant difference between the means of three visits and intervention given. The Tukey’s HSD test was employed as the post hoc test. To compare the mean plaque scores within each group during follow up visits Repeated Measures ANOVA was used. Bonferroni’s post hoc was used as there were many independent or dependent statistical tests applied in this study at the same time. The missing data was imputed using Last Observation Carried Forward (LOCF) method.

## Results

**Figure 1:**
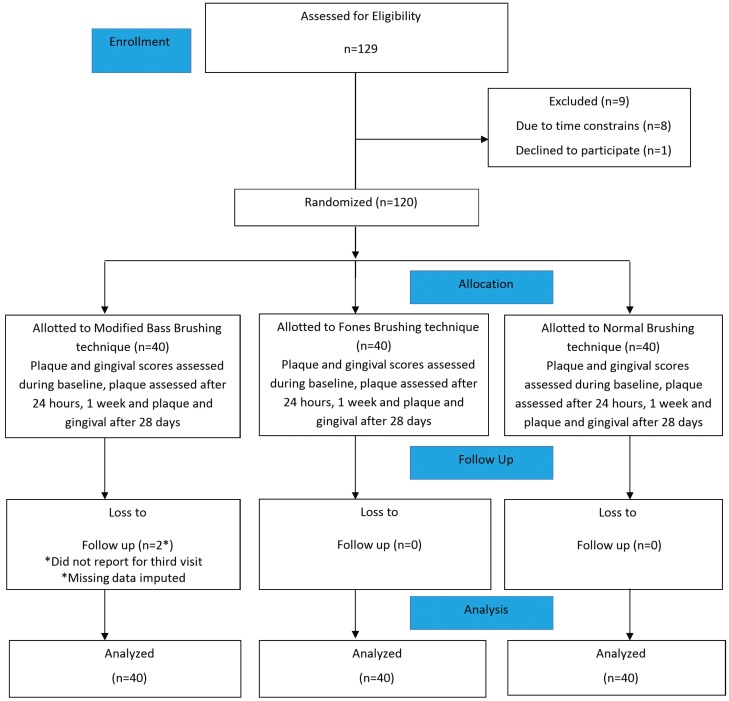
CONSORT Flow chart.

The study was conducted during the period of April 2016 to November 2017. A total of 120 participants were enrolled for the trial. Two participants did not complete their follow up visits (after 28 days). However, they were included in the analysis and the missing values were imputed using Last Observation Carried Forward (LOCF) method (20). The Consolidated Standards of Reporting Trials (CONSORT) diagram for the trial is given (Fig. 1). The mean age of study participants were 22 years and females comprised of 66.6% of the study sample.

At baseline visit, the mean plaque scores measured were 0.74 ± 0.39, 0.77 ± 0.34 and 0.98 ± 0.36 for Modified Bass, Fones and Normal Brushing technique respectively. After 24 hours without any oral hygiene activity the plaque scores increased to 1.04 ± 0.30, 1.11 ± 0.32 and 1.21 ± 0.40. After 1 week following the intervention, the mean plaque score decreased to 0.78 ± 0.36 and 1.03 ± 0.43. After 28 days of using the intervention the plaque scores increased to 1.13 ± 0.44, 1.14 ± 0.40 and 1.08 ± 0.34 ( Table 1).

**Table 1:**
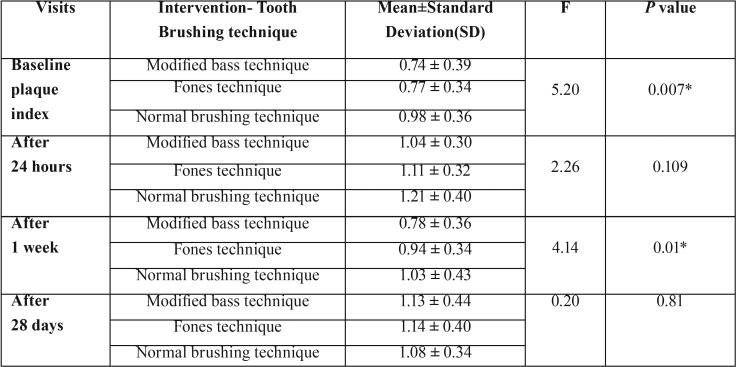
Mean plaque scores of study participants of different groups during each visit.

A difference (*P* value=0.007) was observed in plaque scores at the baseline when comparing three groups with the normal tooth brushing group having highest plaque scores. This was also noted (*P* value =0.01) after one week of intervention following the prescribed brushing method. There was no significant difference between the three groups after 24 hours without brushing (*P* value=0.10) and 28 days following the prescribed brushing method (*P* value=0.81).

A Tukey’s post hoc test showed that during baseline visit, when Modified Bass brushing technique is compared with Fones and Normal brushing, the Normal brushing technique shows statistically significant result (0.009) and when Fones brushing technique is compared with Modified Bass and Normal brushing, again the Normal brushing technique shows statistically significant result (0.03) in reducing the amount of plaque. After 24 hours and 28 days without any oral hygiene activity there was no significant difference between the brushing techniques. During 1 week of using the intervention, when the Modified Bass brushing technique is compared with Fones and Normal brushing, the Normal brushing shows statistically significant (0.01) in reducing the amount of plaque.

 Table 2 shows there was a statistical significant difference within the group in Modified Bass brushing technique (<0.01), Fones Brushing technique (<0.01) and Normal Brushing technique (0.001).

**Table 2:**
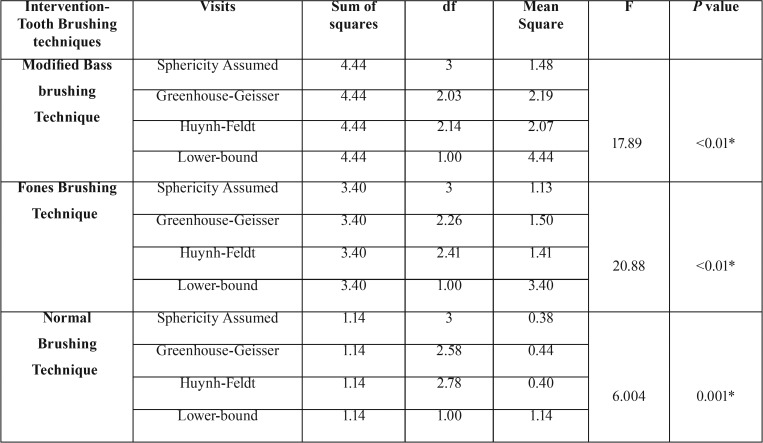
Comparison of each plaque scores within each group during follow up visits.

A Bonferroni’s post hoc test revealed that in the Modified Bass brushing technique, there was a significant difference after 24 hours without using any oral hygiene measures (<0.01) and after 28 days (<0.01) when compared with baseline visits. After 24 hours without any oral hygiene activity there was a significant difference observed after 1 week (<0.01) and after 1 week of using the intervention (tooth brushing technique) there was a significant difference after 28 days (<0.01). In Fones brushing technique when the baseline visit is compared with visits after 24 hours, after 1 week and after 28 days, there was a significant difference in all the visits i.e. After 24 hours (<0.01), after 1 week (<0.01) and 28 days (0.001) of using the intervention. In Normal brushing technique, there was a significant difference when baseline visit is compared after 24 hours (<0.01). There was also a significant difference after 24 hours without any oral hygiene measures when compared after 1 week (0.002).

 Table 3 shows the comparison of mean gingival scores between groups during each visit and there was no significant difference in the baseline and after 28 days. There was no significant difference in baseline and after 28 days with different brushing techniques.

**Table 3:**
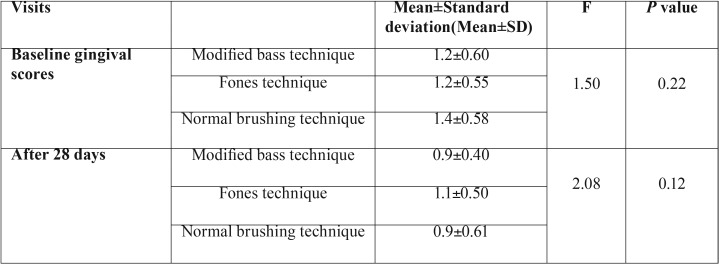
Mean gingival scores of study participants of different groups during each visit.

Mean gingival scores within the group are outlined in  Table 4 shows there was a significant difference in Modified Bass (0.03) and Normal brushing technique (0.01) in baseline and after 28 days.

**Table 4:**
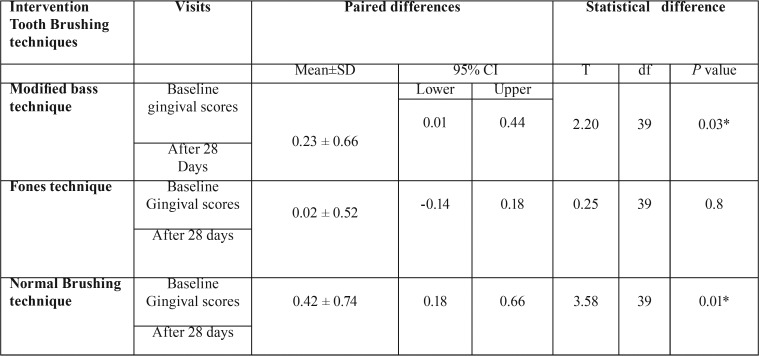
Comparison of mean gingival scores within each group during follow up visits.

## Discussion

A significant reduction in the amount of plaque scores was observed in each of the brushing technique. This finding is in accordance with numerous studies conducted on brushing techniques. One study shows a significant reduction in the amount of plaque in Modified Bass technique after 1 week and 21 days of follow up (5) while another similar study shows a reduction in the amount of dental plaque after 1week of follow up with the Modified Bass brushing technique (21). Bergenholtz *et al.* showed that Bass method was superior in removing plaque from the inaccessible surfaces (22). However, in a study by Hernacke *et al.*, Modified Bass was found to cause no significant differences in plaque scores after 6 weeks.

The anti-plaque efficacy of Fones technique has also been well documented. A 6 week follow up study showed significant differences in plaque scores after using Fones method of brushing (19). As opposed to Modified Bass technique, Fones technique showed a significant difference in the previously mentioned study by Hernacke *et al.* (19).

The Normal (or scrub) method of tooth brushing has also been extensively studied. Muller-Bulla and Courson (23) conducted a systematic review and found that the Horizontal technique (Normal Brushing technique) was found to be the most effective in plaque removal (9-11). However, one of the inherent limitations of this method is the decreased cleansing capacity in proximal surfaces of permanent teeth which can result in gingival recession (24).

The absence of any significant change noted in the follow-up period of 1 month after plaque build-up suggests that the enthusiasm of learning a new technique would have “weaned off” over time. Though participants were provided with a brochure (pamphlets) depicting the steps in performing the new techniques with a view to increase compliance, this might have been too little an effort to teach a completely new technique (19).

With regard to the comparison of efficacy of various techniques, observed results show that the Modified Bass method showed promising short-term outcomes (viz. 1 week) of plaque reduction. Fones technique was almost similar to the Normal Brushing technique. However, the results were insignificant.

Previous literature has also been tilted in favor of Modified Bass technique. A study showing the comparison of different tooth brushing techniques for the effectiveness of plaque control, the Modified Bass brushing technique was found to be effective (21). Bergenholtz *et al.* Also, compared the Bass method with three other techniques: the Roll method, the circular scrub and the horizontal scrub showing that the Bass method was superior to other three techniques in removing supragingival plaque from the lingual surfaces (22).

One of the reasons for this observation could probably be due to Hawthorne effect which shows the increased use of new brushing technique (Modified Bass and Fones technique). Since Fones was also a new technique to the participants, instructions were advised and taught, but there was no significant difference observed. The probable reason might be ineffective inter dental plaque removal (3). Behavioural changes may also be a reason for the participants not implementing or following the method during follow up visits. A study from cognitive brain research have shown that it is essential to imagine or visualize a motion before learning and this requires substantially more cognitive performance (25).

It was observed that there was a significant reduction in gingival index within Modified Bass and Normal brushing technique groups. However, there was no significant reduction in the Fones method. In comparison, of the three techniques, no one technique shows significantly superior results than others. However, this was in contrast to the study done by Hernacke *et al.*; who observed that gingivitis reported significant differences between Fones and control groups after 6 weeks which reached a maximum after 28 weeks (19). Another study showed over the 6-month period, there were significant reduction from baseline in gingival index in two groups (Horizontal Scrub and Modified Bass technique) (26).

The limitation of the study was the use of 24 hours of no oral hygiene activity. Though the ideal plaque maturation time ranges from 48 – 72 hours, the same could not be done due to personal and cultural reasons (27). As the study population was restricted to students, external validity of our study was considered as a limitation. The changes that were observed during the initial phases of the study (1 week) did not translate to the final follow up visit (1 month). In such a scenario, the effect of Hawthorne bias emerging as a result of special attention received, could not be under-estimated (6).

## Conclusions

It was observed that there was as a significant reduction in the amount of plaque among the three brushing techniques. The results showed that the Modified Bass method has some promising short-term outcomes (viz. 1 week) of plaque reduction, but unable to sustain long term effect. However there was no significant difference between the groups was observed.
